# Case report: successful response to bevacizumab combined with erlotinib for a novel *FH* gene mutation hereditary leiomyoma and renal cell carcinoma

**DOI:** 10.3389/fphar.2024.1373020

**Published:** 2024-06-21

**Authors:** Xiaoming Bai, Dan Xiang, Mengxi Huang, Yitian Chen

**Affiliations:** ^1^ Department of Medical Oncology, Jinling Hospital, Nanjing University of Chinese Medicine, Nanjing, Jiangsu, China; ^2^ Department of Medical Oncology, Affiliated Jinling Hospital, Medical School of Nanjing University, Nanjing, China

**Keywords:** hereditary leiomyoma and renal cell carcinoma, *FH* mutation, peripheral blood genetic testing, targeted therapy, bevacizumab combined with erlotinib

## Abstract

*FH*-deficient Renal Cell Carcinoma (*FH*-deficient RCC) are inherited tumors caused by mutations in the fumarate hydratase (*FH*) gene, which plays a role in the tricarboxylic acid cycle. These mutations often result in aggressive forms of renal cell carcinoma (RCC) and other tumors. Here, we present a case of *FH*-deficient RCC in a 43-year-old woman with a history of uterine fibroids. She exhibited a new heterozygous mutation in exon six of the *FH* gene (c.799_803del, c.781_796del). The patient had multiple bone metastases and small subcutaneous nodules in various areas such as the shoulders, back, and buttocks. Biopsy of a subcutaneous nodule on the right side revealed positive expression of 2-succinate-cysteine (2SC), and *FH* staining indicated *FH* expression deletion. The patient underwent treatment with a combination of erlotinib and bevacizumab, which resulted in significant efficacy with moderate side effects. This treatment combination may be recommended as a standard regimen. This case underscores the importance of genetic testing in patients with advanced renal cancer to enhance diagnostic accuracy. Furthermore, it provides insights into potential treatment approaches for *FH*-deficient RCC.

## Introduction

Hereditary leiomyomatosis and renal cell carcinoma (HLRCC) is a kind of hereditary disease caused by germline mutation of fumarate hydratase (*FH*) gene, which is manifested as renal malignant tumor of skin and uterine smooth muscle myoma. *FH*-deficient renal cell carcinoma (RCC) is associated with HLRCC syndrome, which is characterized by *FH* germline mutation or bi-allelic cell *FH* deletion without germline mutation. *FH* system mutation may also lead to renal cell carcinoma. And it has very similar biological functions to HLRCC caused by *FH* germline mutation (Lau et al., 2020).

*FH*-deficient RCC is aggressive, and patients may develop metastatic diseases. Therefore, when diagnosed with *FH*-deficient renal cell carcinoma, timely surgical treatment should be performed to prevent the occurrence of metastatic cancer (Ohe et al., 2018).

*FH* is an enzyme involved in the tricarboxylic acid cycle, facilitating the conversion of fumarate to L-malate. Heterozygous mutations in the *FH* gene can lead to *FH*-deficient RCC, predisposing individuals to aggressive forms of renal cell carcinoma and other tumors (Zyla and Hodgson, 2021). *FH*-deficient RCC typically carries a poor prognosis, with metastatic *FH*-deficient RCC often showing resistance to conventional therapies, necessitating exploration of novel treatment modalities.

The morphological diagnosis of *FH*-deficient RCC is difficult. Immunohistochemistry (IHC) is used to detect the deletion of *FH* expression in tumor cells to diagnose *FH*-deficient renal cell carcinoma, which has been proved to be closely related to the inactivation mutation of the *FH* gene (Smith et al., 2016). In addition, the positive rate of 2SC in *FH*-deficient renal cell carcinoma was 100%, and the positive manifestations were strong positive in diffuse nucleus and cytoplasm, which could be used for auxiliary diagnosis of *FH*-deficient renal cell carcinoma (Muller et al., 2018).

In addition, in order to determine whether patients have metastatic carcinoma, the immunohistochemistry of PAX8, CD10 and Vimentin plays an important role in the diagnosis of metastatic renal cell carcinoma. Among them, CD10 (renal tubular epithelial enzyme) is a common marker of renal cell carcinoma, which can help determine the presence of renal cell carcinoma in immunohistochemical staining (Sangoi et al., 2010). Vimentin is an intermediate filament protein that is associated with metastasis of renal cell carcinoma (Yao et al., 2020).

Here, we present a case of renal cell carcinoma deficient in *FH* in a 43-year-old woman, who harbored a novel heterozygous variant in the sixth exon of the *FH* gene (c.799_803del, c.781_796del). Treatment with a combination of erlotinib and bevacizumab resulted in remarkable efficacy. The successful outcome of this case offers promising insights into HLRCC treatment strategies. Given the limited effective systemic treatments available for *FH*-associated RCC, further investigation into the combination of bevacizumab and erlotinib in a larger patient cohort is warranted.

## Case report

**FIGURE 1:**
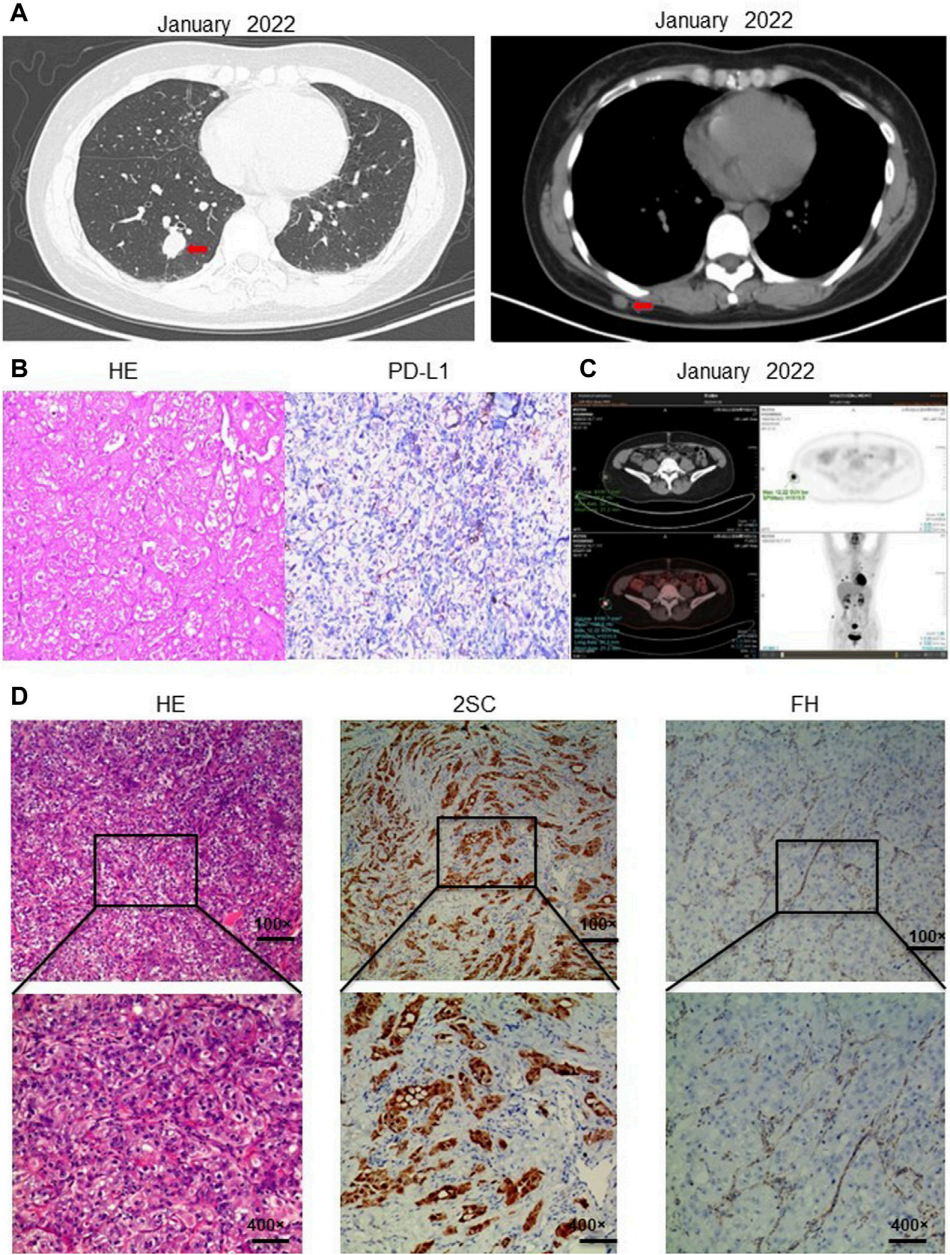
Diagnosis of hereditary smooth muscle tumor and renal cell carcinoma. **(A)** CT in January 2022 showed that the patient had subcutaneous nodules in both lungs and a subcutaneous nodule on the right back. **(B)** Immunohistochemistry showed PD-L1 expression TPS positive, TPS = 5%; PD-L1 expression CPS positive, CPS = 6. **(C)** PET-CT showed cystic lesions in the right kidney; unequal-sized nodules in both lungs; localized bone destruction and uneven density in the left scapula, part of the concha and adnexa, sacrum, and right ilium; bone metastasis was considered. **(D)** Immunohistochemistry showed that the metastatic tumor cells lacked the expression of 2SC; Hematoxylin-eosin staining showed that the metastatic tumor cells had large nuclei with obvious phagocytic nuclei and obvious halos around the nuclei; immunohistochemistry showed that the metastatic tumor cells lacked the expression of fumarate hydratase FH, which supported the diagnosis of renal cell carcinoma with FH deficiency. Information of antibodies for PD-L1, FH, 2SC is shown in Supplementary Table S2.

The patient, a 43-year-old middle-aged woman with a history of uterine fibroids, noticed a subcutaneous nodule on her back in February 2022, measuring approximately 2 cm × 2 cm. The nodule felt hard and was non-tender upon palpation. Computed tomography (CT) scans revealed small subcutaneous nodules on her right back and right buttock, along with variable-sized nodules in both lungs, suggestive of metastasis (Figure 1A). An excisional biopsy of the subcutaneous nodule on her right back was conducted, and immunohistochemistry results indicated characteristics consistent with metastatic cancer: Ckpan (+), Villin (−), CK20(−), CK7(−), P40 (−), Vim (−), Ki67(45%+), S100 (−), SOX10 (−), HMB45(−), TFE3 (−), DES (−), PLAP (−), SALL4 (−), CD10 (focal+), PAX8 (2+), AR (−). Subsequently, another excisional biopsy was performed on a subcutaneous nodule on her right posterior dorsal region. Immunohistochemistry revealed high expression of programmed cell death ligand 1 (PD-L1), with a TPS of 5% and a CPS of 6 (Figure 1B). Further evaluation with positron emission tomography (PET)-CT showed a cystic lesion in the right kidney with a thick capsule wall and increased fluorodeoxyglucose (FDG) uptake, consistent with renal carcinoma, with a possibility of cystic adenocarcinoma. Additionally, nodules of unequal sizes in both lungs displayed increased FDG uptake, indicative of metastasis. Localized bone destruction, uneven density, and increased FDG uptake were observed in the left scapula, part of the concha and adnexa, sacrum, and right ilium, suggesting bone metastasis (Figure 1C). Based on the collective imaging and pathological findings, the patient received a final diagnosis of high-grade renal cell carcinoma.

**FIGURE 2:**
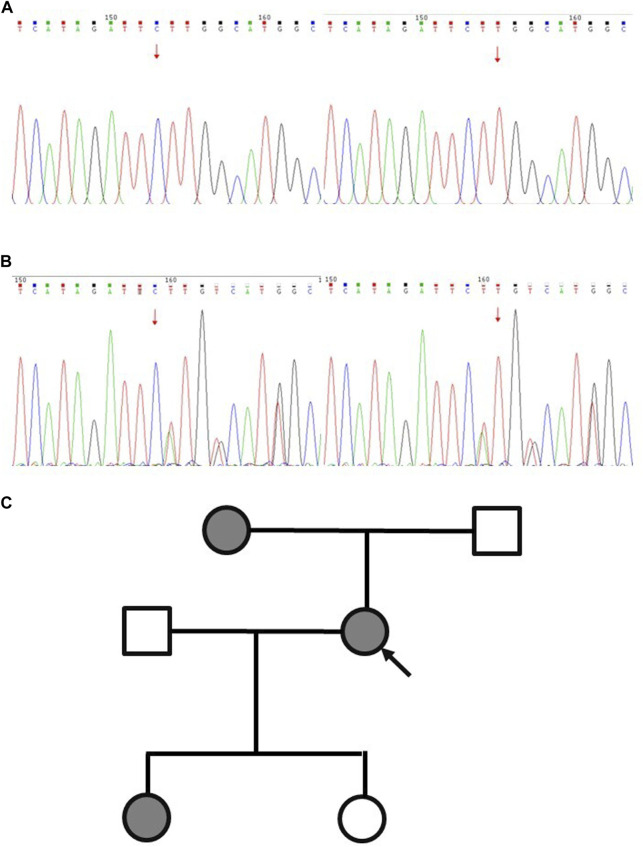
Schematic diagram of Sanger sequencing validation results for the proband and wild-type FH variants. **(A)** Wild type. **(B)** The proband. **(C)** Pedigree of the family with three patients. The black symbols represent the affected members with renal carcinoma, and the arrow indicates the proband.

In February 2023, a resection biopsy of the patient’s right dorsal subcutaneous nodule was conducted due to the persistence of severe subcutaneous nodules 1 year post-treatment. HE staining revealed enlarged nuclei with prominent eosinophilic nucleoli and a clear halo around the nucleolus (Figure 1D). Immunohistochemistry for 2SC demonstrated positive staining (Figure 1D), while staining for *FH* showed loss of *FH* expression (Figure 1D). Following the patient’s informed consent, whole exon sequencing was performed on the patient’s tissue, revealing suspicious pathogenic mutations that could account for the patient’s phenotype. Sequencing results identified heterozygous mutations in the *FH* gene (NM_000143: c.799_803del, p. P267fs; NM_000143: c.781_796del, p. R261fs) (Supplementary Table S1). Integrating the genetic testing and pathological findings, the patient was diagnosed with *FH* genotype-deficient renal cell carcinoma. Given the hereditary nature of *FH* gene-deficient renal cell carcinoma, whole exon gene sequencing was conducted on peripheral blood samples from the patient’s mother and two daughters. Results indicated that the patient’s mother and one daughter harbored the same mutations at the identical sites within the *FH* gene (Figure 2A). Wild-type Sanger sequencing is depicted in Figure 2B. The patient’s family pedigree is illustrated in Figure 2C.

**FIGURE 3:**
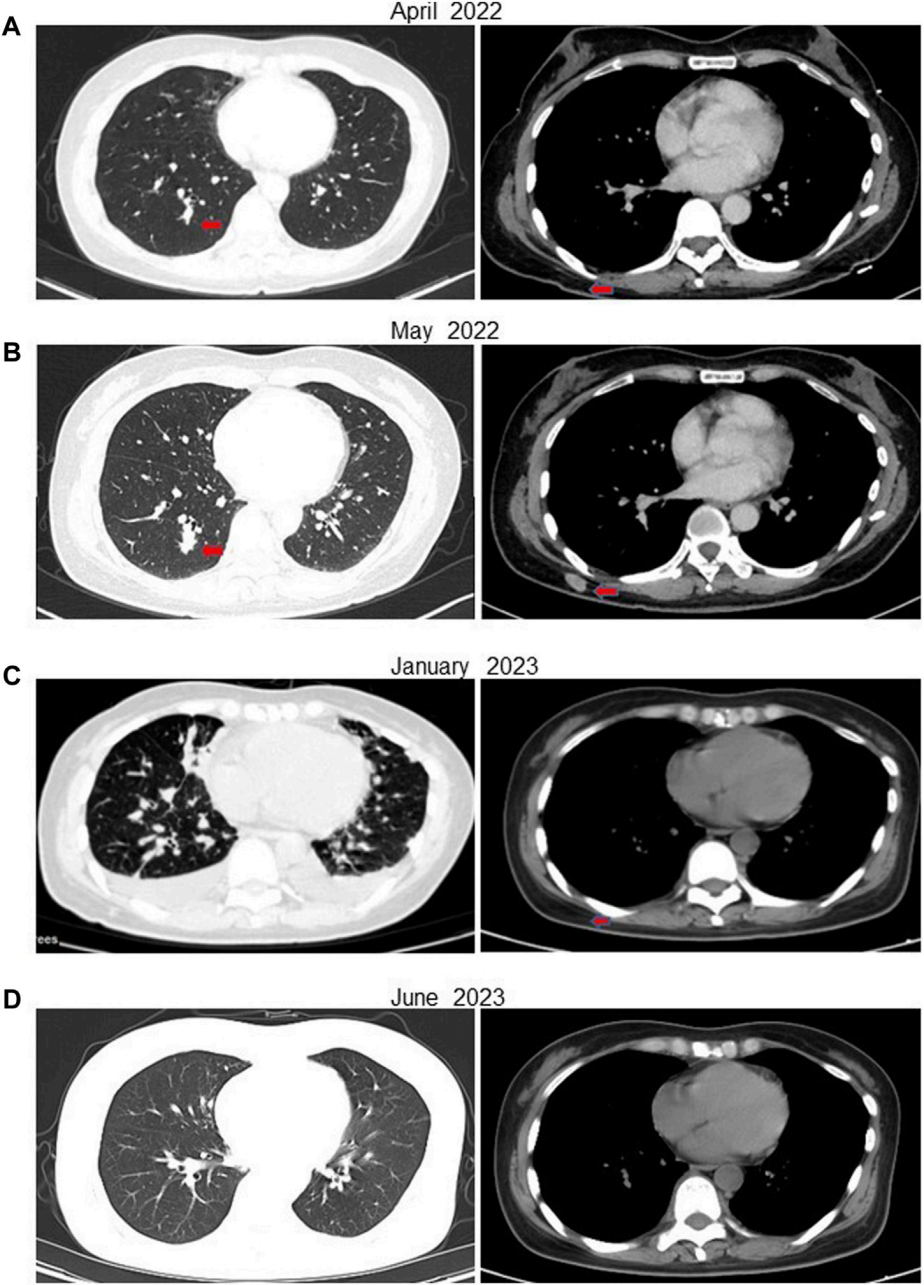
CT of the patient at various stages after receiving treatment. **(A)** In April 2022, after 6 weeks of treatment with pembrolizumab in combination with sunitinib, the patient’s metastases were significantly reduced. **(B)** In May 2022, after 5 weeks of treatment with pembrolizumab in combination with axitinib due to the patient’s intolerance of sunitinib, the subcutaneous nodule on the right side of the back was enlarged compared with the previous one. **(C)** After eight cycles of the original regimen, a CT in January 2023 showed multiple metastases in both lungs that were significantly more advanced than before. **(D)** ACT in July 2023 showed a significant reduction in the patient’s metastatic lesions.

**FIGURE 4:**
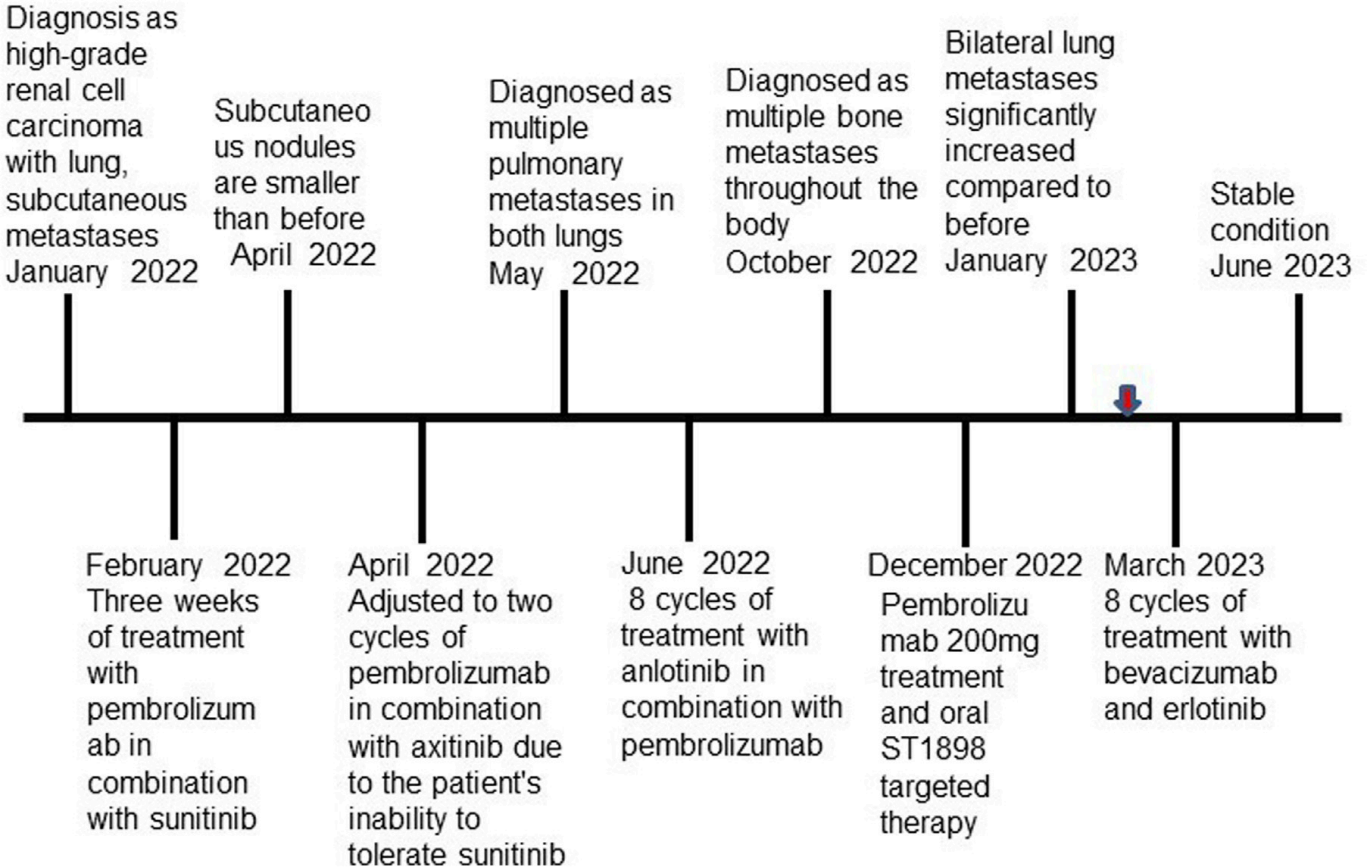
Timeline of the case. Red arrow indicates that the patient was diagnosed with FH-deficient renal cell carcinoma.

In February 2022, following a diagnosis of high-grade renal cell carcinoma, the patient commenced immediate treatment with two cycles of pembrolizumab combined with sunitinib (Pembrolizumab 200mg, every 3 weeks; sunitinib 50 mg, once daily for 2 weeks, with a 1-week break). Subsequent CT scans in April 2022 revealed significant reductions in the size of subcutaneous nodules and pulmonary metastases on the right back compared to previous scans (Figure 3A). However, due to intolerance to sunitinib, the treatment was modified to pembrolizumab combined with axitinib for 2 weeks (Pembrolizumab 200 mg, every 3 weeks; Axitinib 5mg, twice daily). In May 2022, CT scans indicated an increase in multiple small nodules in various subcutaneous areas and an increase in metastatic tumors in both lungs compared to April 2022 (Figure 3B). Subsequently, the patient underwent treatment with anlotinib in combination with pembrolizumab for eight cycles (Pembrolizumab 200 mg, every 3 weeks; Anlotinib 12 mg, once daily for 2 weeks, with a 1-week break). PET-CT results in October 2022 demonstrated significant progression of bone metastases throughout the body compared to May 2022 (Supplementary Figure S1A). In December 2022, the patient received treatment with pembrolizumab alongside oral ST1898 targeted therapy. However, a CT scan in January 2023 revealed significant enlargement of bilateral lung metastases compared to October 2022 (Figure 3C). Subsequently, in February 2023, following the diagnosis of *FH*-deficient renal cell carcinoma, the patient’s treatment regimen was adjusted. Treatment with pembrolizumab, erlotinib, and bevacizumab was initiated, although immunization was temporarily suspended due to significantly increased pituitary prolactin levels. In March 2023, the patient underwent eight cycles of treatment with bevacizumab and erlotinib. A CT reexamination in June 2023 showed a significant reduction in metastatic lesions, with the patient’s condition stabilized (Figure 3D). The timeline of the case is illustrated in Figure 4, with the top axis depicting the diagnostic process and the bottom axis showing the treatment process. Consent for publication of this case report was obtained from the patient.

## Discussion

HLRCC is an autosomal dominant genetic disorder linked to inactivating mutations in the *FH* gene. Typically, individuals with HLRCC exhibit a genetic predisposition to skin and uterine leiomyomas, as well as kidney tumors (Linehan and Ricketts, 2019). The *FH* gene mutation leads to dysfunction or structural abnormalities in the *FH* protein, which plays a crucial role in catalyzing the conversion of fumarate to malate within the tricarboxylic acid (TCA) cycle—a fundamental process in cellular energy metabolism. Disruption of this enzymatic activity due to the *FH* mutation results in fumarate accumulation and decreased malate levels within cells. This perturbation in the TCA cycle adversely affects cellular energy metabolism and ATP production. Moreover, the *FH* mutation may induce excessive free radical generation, thereby promoting cellular oxidative stress, apoptosis, and potentially contributing to tumor development (Valcarcel-Jimenez and Frezza, 2023). *FH* serves as a pivotal metabolic enzyme in the TCA cycle, and its deficiency leads to intracellular fumarate accumulation. Fumarate buildup within mitochondria and subsequent leakage into the cytoplasm, termed “tumor metabolites,” is associated with the development of skin leiomyomas, uterine fibroids, and kidney cancer (Lindner et al., 2022). HLRCC represents a subtype of RCC characterized by notable invasiveness, predominantly affecting young individuals and often accompanied by early metastasis (Yu et al., 2021). Concurrently, intracellular fumarate accumulation can induce a stable chemical modification of intracellular proteins known as abnormal succinylation. The presence of modified proteins can be detected using 2SC antibodies. While immunohistochemical detection of *FH* protein remains crucial for diagnosing HLRCC, some HLRCC tumor cells may still express *FH* protein. Therefore, combined detection of *FH* and 2SC can enhance the diagnostic accuracy of HLRCC (Zheng et al., 2023).

A recent study documented a case of HLRCC in which a patient remained free of tumor recurrence or metastasis for 24 months following treatment with a PD-1 inhibitor, Pembrolizumab (Wang et al., 2021). PD-1 inhibitors have emerged as the preferred therapeutic option for many cases of RCC (McDermott et al., 2018; Aggen et al., 2020; Brown et al., 2020). PD-1 is expressed on B cells, T cells, and regulatory T cells, and its expression is indicative of T-cell exhaustion. PD-L1, found to be upregulated in both hemangiomas and solid tumors, acts as a checkpoint molecule that inhibits the host’s anti-tumor immunity (Jiang et al., 2019). Consequently, inhibitors targeting PD-1 and PD-L1 have been employed in tumor treatment (Shi et al., 2011). Research findings suggest that PD-L1 expression is prevalent in the majority of HLRCC cases, rendering immunotherapy a promising therapeutic avenue for HLRCC (Sun et al., 2021). Moreover, elevated expression of PD-L1 has been observed in the subcutaneous metastases of patients discussed in our reported case. Therefore, it is imperative to assess the immune microenvironment, including PD-L1 expression and CD8^+^ T cells, in HLRCC. Such evaluations can provide valuable insights to guide the development of more precise clinical treatment strategies.

In addition, we evaluated the pathogenicity of *FH* gene defects in patients, including the following aspects: Gene mutation analysis; through sequencing and analysis of the *FH* gene, deletion mutations with the *FH* gene (c.799 _ 803del, c.781 _ 796del) can be detected. Determination of enzyme activity; the expression of *FH* gene was determined by immunohistochemistry. The patient’s immune results showed that the expression of *FH* gene was missing. Based on the above evaluation results, the pathogenicity of *FH* gene defects can be determined, and corresponding diagnosis and treatment suggestions can be provided for patients.

*FH*-RCC is relatively rare, posing challenges in standardized diagnosis due to the lack of data from multicenter clinical trials with large sample sizes. Real-world treatment outcomes exhibit considerable heterogeneity, and there is a lack of uniform standardized treatment protocols. In this context, we present a case of an HLRCC-RCC patient with a history of uterine fibroids, wherein gene testing revealed a heterozygous mutation in the *FH* gene. The patient underwent treatment with a combination of bevacizumab and erlotinib, resulting in symptom relief. The combination of bevacizumab and erlotinib is a strategy to enhance the anti-tumor effect of drugs based on two different mechanisms. Bevacizumab is an anti-vascular endothelial growth factor (VEGF) monoclonal antibody, which can selectively bind to human vascular growth factor (VEGF) and block its biological activity. It can inhibit the binding of VEGF to its receptors VEGFR-1 and VEGFR-2 located on endothelial cells, so that VEGF loses its biological activity and reduces tumor angiogenesis, thus inhibiting tumor growth (Garcia et al., 2020). Erlotinib is a targeted therapy drug, which belongs to the epidermal growth factor receptor (EGFR) tyrosine kinase inhibitor (TKI) class, by inhibiting the activity of EGFR, thereby preventing the growth and spread of tumor cells. EGFR is a protein expressed on the surface of tumor cells, which can promote the growth and survival of tumor cells. Erlotinib can bind to EGFR and block its activity, thereby inhibiting the growth and spread of tumor cells (Grépin et al., 2020). The combination of bevacizumab and erlotinib, abbreviated as the E-B regimen, has shown efficacy in treating *FH*-deficient RCC (Carril-Ajuria et al., 2021). The main purpose of the combination of these two drugs is to enhance the anti-tumor effect through two different mechanisms. This combined effect can theoretically improve the therapeutic effect and is expected to reduce the development of drug resistance. The results of first-line treatment showed that the objective remission rate of *FH*-deficient RCC patients treated with E-B regimen was 50%, the median progression-free survival was 13.3 months, and the disease control rate was 90% (Zhou et al., 2021). The successful outcome of this case may offer novel insights into the treatment of *FH*-deficient RCC, suggesting the potential utility of the E-B regimen in managing this condition.

## Conclusion

In this case report, the patient’s diagnosis of *FH*-deficient RCC was delayed due to the lack of prompt genetic testing. *FH*-deficient RCC involves a mutation in the *FH* gene, and genetic testing holds significant importance for its treatment. The patient exhibited a novel heterozygous mutation (c.799_803del, c.781_796del) in the sixth exon of the *FH* gene. Following treatment with a combination of bevacizumab and erlotinib, metastases decreased or disappeared, leading to disease stabilization. This underscores the necessity of genetic testing for patients and their relatives with advanced RCC, aiding in the early detection of *FH*-deficient RCC and facilitating appropriate treatment. The treatment approach employed in this case offers insights for managing *FH*-deficient RCC.

## Data Availability

The original contributions presented in the study are included in the article/Supplementary Material, further inquiries can be directed to the corresponding authors.

## Supplementary material

SUPPLEMENTARY TABLE S1
Gene mutation information of the patient.

SUPPLEMENTARY TABLE S2
Information of antibodies for PD-L1, FH, 2SC.

## References

[B1] Aggen D. H. Drake C. G. Rini B. I. (2020). Targeting PD-1 or PD-L1 in metastatic kidney cancer: combination therapy in the first-line setting. Clin. Cancer Res. 26 (9), 2087–2095. 10.1158/1078-0432.CCR-19-3323 31948999

[B2] Brown L. C. Desai K. Zhang T. Ornstein M. C. (2020). The immunotherapy landscape in renal cell carcinoma. BioDrugs 34 (6), 733–748. 10.1007/s40259-020-00449-4 33048299

[B3] Carril-Ajuria L. Colomba E. Cerbone L. Romero-Ferreiro C. Crouzet L. Laguerre B. (2021). Response to systemic therapy in fumarate hydratase–deficient renal cell carcinoma. Eur. J. Cancer 151, 106–114. 10.1016/j.ejca.2021.04.009 33975058

[B4] Garcia J. Hurwitz H. I. Sandler A. B. Miles D. Coleman R. L. Deurloo R. (2020). Bevacizumab (Avastin®) in cancer treatment: a review of 15 years of clinical experience and future outlook. Cancer Treat. Rev. 86, 102017. 10.1016/j.ctrv.2020.102017 32335505

[B5] Grépin R. Guyot M. Dumond A. Durivault J. Ambrosetti D. Roussel J.-F. (2020). The combination of bevacizumab/Avastin and erlotinib/Tarceva is relevant for the treatment of metastatic renal cell carcinoma: the role of a synonymous mutation of the EGFR receptor. Theranostics 10 (3), 1107–1121. 10.7150/thno.38346 31938054 PMC6956821

[B6] Jiang Y. Chen M. Nie H. Yuan Y. (2019). PD-1 and PD-L1 in cancer immunotherapy: clinical implications and future considerations. Hum. Vaccines Immunother. 15 (5), 1111–1122. 10.1080/21645515.2019.1571892 PMC6605868 30888929

[B7] Lau H. D. Chan E. Fan A. C. Kunder C. A. Williamson S. R. Zhou M. (2020). A clinicopathologic and molecular analysis of fumarate hydratase-deficient renal cell carcinoma in 32 patients. Am. J. Surg. Pathology 44 (1), 98–110. 10.1097/PAS.0000000000001372 31524643

[B8] Lindner A. K. Tulchiner G. Seeber A. Siska P. J. Thurnher M. Pichler R. (2022). Targeting strategies in the treatment of fumarate hydratase deficient renal cell carcinoma. Front. Oncol. 12, 906014. 10.3389/fonc.2022.906014 35912170 PMC9337267

[B9] Linehan W. M. Ricketts C. J. (2019). The Cancer Genome Atlas of renal cell carcinoma: findings and clinical implications. Nat. Rev. Urol. 16 (9), 539–552. 10.1038/s41585-019-0211-5 31278395

[B10] McDermott D. F. Huseni M. A. Atkins M. B. Motzer R. J. Rini B. I. Escudier B. (2018). Clinical activity and molecular correlates of response to atezolizumab alone or in combination with bevacizumab versus sunitinib in renal cell carcinoma. Nat. Med. 24 (6), 749–757. 10.1038/s41591-018-0053-3 29867230 PMC6721896

[B11] Muller M. Guillaud-Bataille M. Salleron J. Genestie C. Deveaux S. Slama A. (2018). Pattern multiplicity and fumarate hydratase (FH)/S-(2-succino)-cysteine (2SC) staining but not eosinophilic nucleoli with perinucleolar halos differentiate hereditary leiomyomatosis and renal cell carcinoma-associated renal cell carcinomas from kidney tumors without FH gene alteration. Mod. Pathol. 31 (6), 974–983. 10.1038/s41379-018-0017-7 29410489

[B12] Ohe C. Smith S. C. Sirohi D. Divatia M. de Peralta-Venturina M. Paner G. P. (2018). Reappraisal of morphologic differences between renal medullary carcinoma, collecting duct carcinoma, and fumarate hydratase–deficient renal cell carcinoma. Am. J. Surg. Pathology 42 (3), 279–292. 10.1097/PAS.0000000000001000 PMC8015937 29309300

[B13] Sangoi A. R. Karamchandani J. Kim J. Pai R. K. McKenney J. K. (2010). The use of immunohistochemistry in the diagnosis of metastatic clear cell renal cell carcinoma: a review of PAX-8, PAX-2, hKIM-1, RCCma, and CD10. Adv. Anatomic Pathology 17 (6), 377–393. 10.1097/PAP.0b013e3181f89400 20966644

[B14] Shi F. Shi M. Zeng Z. Qi R.-Z. Liu Z.-W. Zhang J.-Y. (2011). PD-1 and PD-L1 upregulation promotes CD8+ T-cell apoptosis and postoperative recurrence in hepatocellular carcinoma patients. Int. J. Cancer 128 (4), 887–896. 10.1002/ijc.25397 20473887

[B15] Smith S. C. Trpkov K. Chen Y.-B. Mehra R. Sirohi D. Ohe C. (2016). Tubulocystic carcinoma of the kidney with poorly differentiated foci: a frequent morphologic pattern of fumarate hydratase-deficient renal cell carcinoma. Am. J. Surg. Pathology 40 (11), 1457–1472. 10.1097/PAS.0000000000000719 PMC5577927 27635946

[B16] Sun G. Zhang X. Liang J. Pan X. Zhu S. Liu Z. (2021). Integrated molecular characterization of fumarate hydratase–deficient renal cell carcinoma. Clin. Cancer Res. 27 (6), 1734–1743. 10.1158/1078-0432.CCR-20-3788 33414138

[B17] Valcarcel-Jimenez L. Frezza C. (2023). Fumarate hydratase (FH) and cancer: a paradigm of oncometabolism. Br. J. Cancer 129 (10), 1546–1557. 10.1038/s41416-023-02412-w 37689804 PMC10645937

[B18] Wang T. Huang Y. Huang X. Lv Z. Tian S. Ma X. (2021). Complete response of hereditary leiomyomatosis and renal cell cancer (HLRCC)-Associated renal cell carcinoma to pembrolizumab immunotherapy: a case report. Front. Oncol. 11, 735077. 10.3389/fonc.2021.735077 34722283 PMC8554149

[B19] Yao J. x. Chen X. Zhu Y. j. Wang H. Hu X. y. Guo J. m. (2020). Prognostic value of Vimentin is associated with immunosuppression in metastatic renal cell carcinoma. Front. Oncol. 10, 1181. 10.3389/fonc.2020.01181 32850341 PMC7417332

[B20] Yu Y. Zheng M. Zhu W. Zhao F. Guan B. Shen Q. (2021). Hereditary leiomyomatosis and renal cell cancer (HLRCC): case series and review of the literature. Urologic Oncol. Seminars Orig. Investigations 39 (11), 791.e9–791.e16. 10.1016/j.urolonc.2021.07.026 34462205

[B21] Zheng L. Zhang X. Pan X. Huang Z. Zhang M. Xian J. (2023). AKR1B10 is a new sensitive and specific marker for fumarate hydratase-deficient renal cell carcinoma. Mod. Pathol. 36 (11), 100303. 10.1016/j.modpat.2023.100303 37580017

[B22] Zhou Q. Xu C.-R. Cheng Y. Liu Y.-P. Chen G.-Y. Cui J.-W. (2021). Bevacizumab plus erlotinib in Chinese patients with untreated, EGFR-mutated, advanced NSCLC (ARTEMIS-CTONG1509): a multicenter phase 3 study. Cancer Cell 39 (9), 1279–1291.e3. 10.1016/j.ccell.2021.07.005 34388377

[B23] Zyla R. E. Hodgson A. (2021). Gene of the month: FH. J. Clin. Pathology 74 (10), 615–619. 10.1136/jclinpath-2021-207830 34353877

