# Supplementary material for: Case report: successful response to bevacizumab combined with erlotinib for a novel FH gene mutation hereditary leiomyoma and renal cell carcinoma
Source: Front Pharmacol. 2024 Jun 21;15:1373020. doi: 10.3389/fphar.2024.1373020 (PMC11224512; doi:10.3389/fphar.2024.1373020)
Supplement: Supplementary file 2 [file Image1.pdf]

Supplementary Material

Supplementary Figures

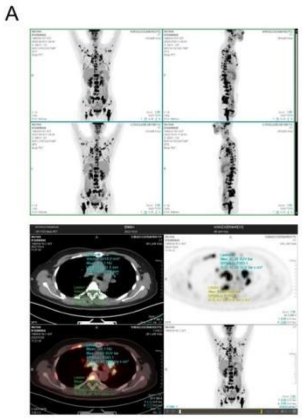

Supplementary Figure1 . Additional computed tomography infermation.

A. In October 2022, PET-CT showed multiple bone metastases throughout the body, with significant progress. Subcutaneous nodules on the right back with increased FDG uptake.
